# Ocular Thermal Burn Injury in the Emergency Department

**DOI:** 10.7759/cureus.7137

**Published:** 2020-02-28

**Authors:** Sheila Bawany, Tracy Macintosh, Latha Ganti

**Affiliations:** 1 Emergency Medicine, University of Central Florida College of Medicine/Hospital Corporation of America Graduate Medical Education Consortium of Greater Orlando, Orlando, USA; 2 Emergency Medicine, Osceola Regional Medical Center, Kissimmee, USA; 3 Emergency Medicine, Envision Physician Services, Orlando, USA; 4 Emergency Medicine, Polk County Fire Rescue, Bartow, USA

**Keywords:** ocular thermal burn, eye injury

## Abstract

We present a case of an ocular thermal burn from a cooking accident where vegetable oil splashed into the patient's face. The emergency department evaluation and management of ocular thermal burns is discussed. Prompt evaluation, copious irrigation, and consultation with ophthalmology are recommended. Teaching points are highlighted.

## Introduction

Ocular burn injuries remain a relatively uncommon complaint in the emergency department (ED). Eye trauma comprises 3% of ED visits, of which only 7% relate to ocular burns. The majority of eye complaints are due to corneal abrasions and foreign bodies. Ocular burn injuries tend to be less common or less severe due to the protective blinking reflexes or due to Bell’s phenomenon if present, wherein the eye rolls upward and outward in response to the eye being opened (also known as palpebral oculogyric reflex) [1]. 

Initial evaluation of a patient determined to have a thermal burn injury includes a complete physical exam with assessment of visual acuity, extraocular motion, intraocular pressure, and fluorescein staining [2]. Patients should also be evaluated for the presence of a foreign body as this may cause further corneal insult. We present a case of ocular thermal burn secondary to a vegetable oil splash.

## Case presentation

A previously healthy 33-year-old woman presented to the ED in the late evening complaining of burns to her face and hands as well as acute ophthalmoplegia and blurred vision. The patient reported that earlier in the evening she was deep frying churros (fried dough) at home when the vegetable oil in the fryer exploded and splashed onto her face and eyes. The patient applied aloe vera to her skin prior to presentation which provided some symptomatic relief.

On physical examination, the patient had superficial burns involving her frontal, maxillary, and mandibular regions of her face. She also had a single non-circumferential second degree burn over the palmar aspect of her right hand. Her superior eyelids were minimally swollen bilaterally and eyelashes were clean and intact. Her extraocular movements were intact, pupils were 3 mm equal round and reactive, and sclera and limbus appeared intact. Her conjunctiva was minimally injected. Her unaided visual acuity was OD: 30/20, OS: 70/20, bilateral: 25/20.

**Figure 1:**
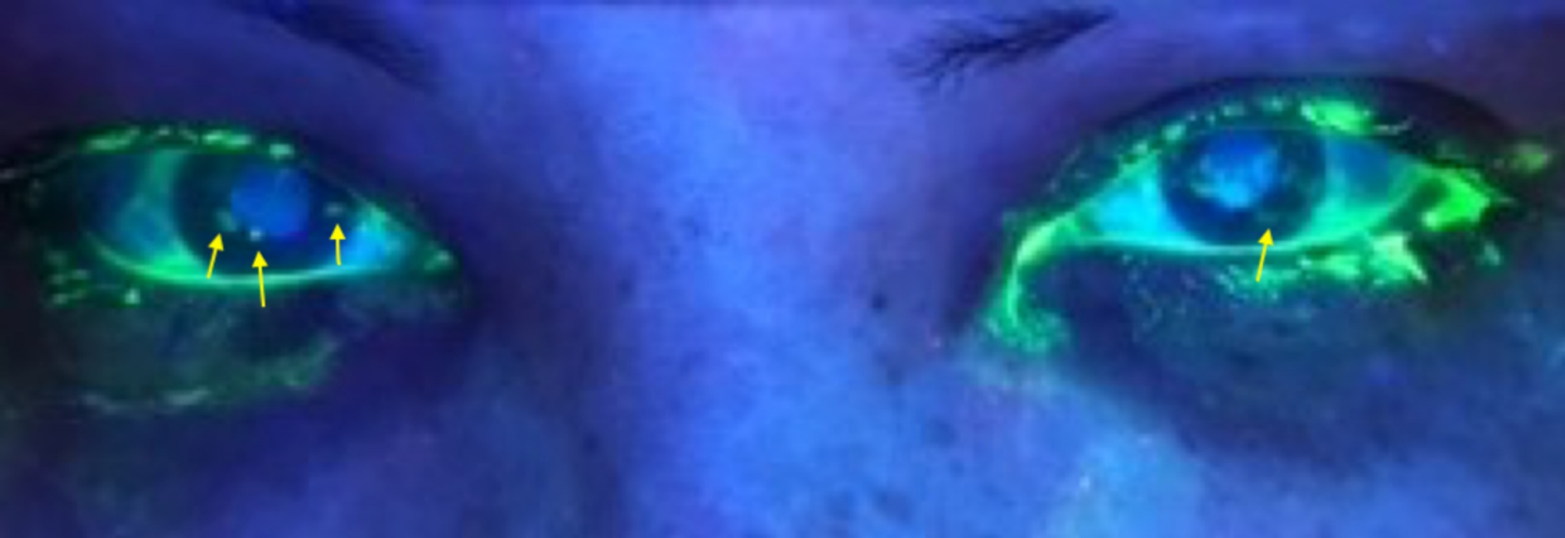
Fluorescein staining showed punctate corneal uptake (arrows).

Fluorescein staining showed punctate corneal uptake as seen in Figure 1.

Her eyes were copiously irrigated with 1 L of lactated Ringer's solution for each eye using a commercial irrigation lens. The patient was discharged home with pain control, a lubricating eye ointment, and erythromycin ointment. Per ophthalmology, the patient was to follow up in their office within 48 hours. 

## Discussion

Despite vegetable oil being an everyday household item, vegetable oil explosion with associated ocular burn is a relatively uncommon presentation in the ED due to the protective blinking reflex and eyes rolling up [1,3]. Thermal burns to the globe rarely result in permanent damage or vision loss. In one retrospective study, it was found that approximately 15% of facial burn injuries were associated with ocular involvement and of that only a small number resulted in serious ocular pathology [4]. The limbus contains corneal epithelial cells. Limbic damage and ischemia can impair normal corneal healing [5].

Management of ocular burns begins at the time of injury with copious irrigation and is continued in the ED with more sophisticated techniques and solutions. The use of a contact lens attached to plastic tubing can help facilitate continuous irrigation. Normal saline is commonly used due to its handiness; it is however a hypotonic solution compared to aqueous humor which may lead to increased corneal uptake. Lactated Ringer's solution on the other hand has similar osmolarity to aqueous humor, and thus may prevent corneal swelling. Irrigation should be generous with no less than 1 L to the affected eye [6].

**Figure 2:**
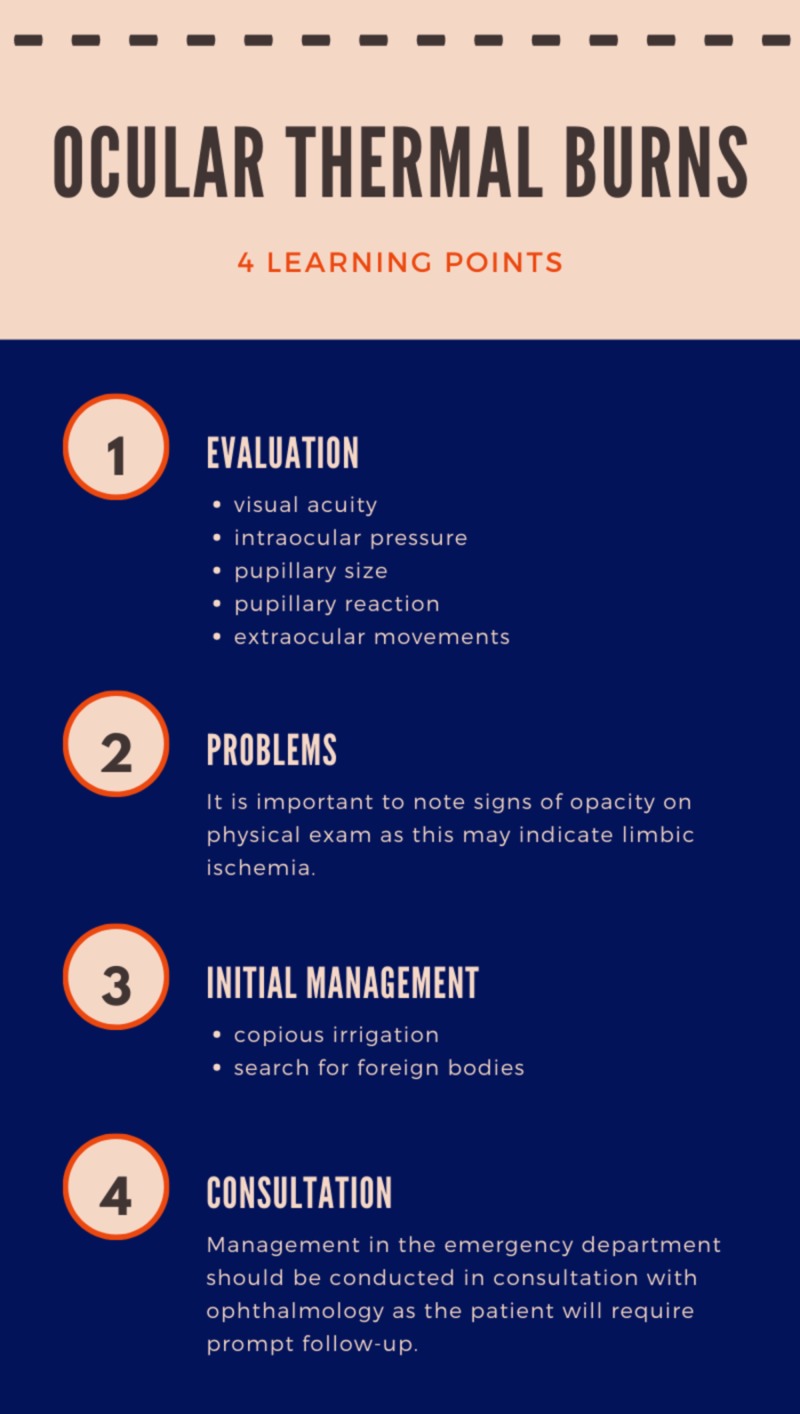
Ocular thermal burns - learning points

Even in the presence of a seemingly uninjured globe, foreign bodies such as food particles or extrinsic projectile materials may remain and may be difficult to detect which highlights the importance of expeditious, copious irrigation in the ED followed by complete eye exam, and fluorescein staining followed by close evaluation with an ophthalmologist (Figure 2).

Thermal ocular burns rarely result in permanent damage or vision loss. Approximately 15% of facial burn injuries are associated with ocular involvement and of that only a small number resulted in serious ocular pathology [7]. One study concluded that of the periocular thermal injuries which do result in corneal injury, up to 87% of cases make a full recovery [8]. In rare cases, corneal burns can result in corneal opacity, requiring corneal transplant [9].

## Conclusions

Initial evaluation and management of corneal burns requires treatment with copious irrigation as well as assessment of visual acuity, pupillary size, pupillary reaction, extraocular movement, and intraocular pressure. It is important to note signs of opacity on physical exam as this may indicate limbic ischemia. Management in the ED should be conducted in consultation with ophthalmology as the patient will require prompt follow-up.
